# Editorial: Gender, sexuality, and well-being: impact on well-being due to gender and sexual orientation-based discrimination

**DOI:** 10.3389/fsoc.2025.1584881

**Published:** 2025-03-19

**Authors:** Ana Álvarez-Muelas, Marta Badenes-Sastre, Patricia Flor-Arasil, Mayra Gomez-Lugo, Martin Sanchez-Gomez

**Affiliations:** ^1^Mind, Brain, and Behavior Research Centre, University of Granada, Granada, Spain; ^2^University of Jaén, Jaén, Andalusia, Spain; ^3^Valencian International University, Valencia, Spain; ^4^Universidad Internacional de La Rioja (UNIR), Logroño, Spain; ^5^Fundación Universitaria Konrad Lorez, Bogotá, Colombia

**Keywords:** gender roles, gender-based violence, sexual attitudes, well-being, discrimination, homophobia, sociology of health

This editorial considers a sociological reflection of how stigmas, taboos, or discrimination related to gender, sexual orientation, identity, or expression can significantly impact our wellbeing. The Research Topic has prompted studies that show how individuals may face distinct negative experiences (such as intimate partner violence or harassment), encounter negative attitudes (homophobia, misogyny, or transphobia), and be subject to various negative behaviors (such as rejection or exclusion) based on their gender or sexuality.

There are eight original research articles within this Research Topic. We briefly outline each of these contributions below.

In the first article, Karniej et al. propose to test the psychometric properties of the Polish-language version of the Gay Affirmative Practice Scale (GAP-PL) in a sample of 329 participants. Analysis of the individual questionnaire items, confirmatory factor analysis, and internal consistency analysis of the GAP scale were performed. The results showed that the scale's internal consistency, reliability, and factor structure are excellent (Cronbach's alpha for subscales between 0.936 and 0.949 and McDonald's omega coefficient of 0.963). The GAP-PL demonstrates adequate properties of factorial validity and can be used in research and clinical practice in Polish-speaking populations.

In the second article, Jaén et al. explored the role of Medical-Legal Partnerships in addressing legal issues that negatively impact the health of people living with HIV in the United States. Using a cross-sectional survey of 111 providers, the study applied a socio-ecological framework to examine the benefits and challenges of integrating legal and medical services to improve HIV care outcomes. Thematic analysis highlighted key advantages, including improved patient health, comprehensive service provision, enhanced staff competence, and potential policy influence. The study has underscored the essential role of these partnerships in reducing structural stigma and discrimination while advocating for policy reforms to promote health equity for people living with HIV.

The article by Seretlo et al. could be participants who identify as queer individuals' perspectives on improving sexual and reproductive healthcare services in Gauteng Province, South Africa. Researchers conducted semi-structured interviews of 22 participants who identified as queer aged 18 and over within a queer-inclusive non-governmental organization. The thematic content analysis identified key themes such as the need for healthcare equity and enhanced training for healthcare providers for the creation of a health system that is more equitable, empathetic, and adapted for everyone. The study concluded by advocating for inclusive policies and further research to address the unique healthcare needs of the queer community.

In the fourth article, Kolié et al. analyzed perceptions, responses, and challenges faced in combating sexual violence in rural Guinea in 2020. All cases of gender violence reported by public health facilities and directorates of girls and women were collected in the health districts of Télimélé and Mamou. Quantitative results indicated that 61% of women who reported gender violence in Mamou presented sexual violence, whereas in Télimélé this figure was only 8%. Qualitative data obtained from 34 in-depth interviews showed several barriers (socio-economic constraints, lack of skilled healthcare providers, frequent stock-outs of essential medical supplies, and absence of psycho-social and legal support at the community level) to accessing health services and providing comprehensive care to survivors.

In the fifth article, Wong et al. examined which variables influence mood disorders including depression, anxiety, and stress among emerging adults. Variables such as age, sex assigned at birth, being LGBTQ+, income, religion and spirituality, and marital satisfaction were explored. Study participants were emerging Filipino adults aged 18 to 29 living in Metro Manila. The results showed that being LGBTQ+ could be a risk factor for depression, anxiety, and stress. It also highlighted other interesting conclusions that arise from the tested variables acting as a protection or risk factor for mental health.

In the next article, Calvillo et al. carried out a systematic review to examine the relationship between intimate partner violence (IPV) and sexual health outcomes in adults over the last decade. The review included 27 articles and follows the PRISMA guidelines and quality assessment. The study found that IPV is associated with poorer sexual health outcomes, including a higher risk of sexually transmitted infections, unintended pregnancies, sexual dysfunction, and lower sexual satisfaction. The review highlighted the need for targeted interventions and culturally sensitive approaches, noting significant research gaps for cisgender heterosexual men, men who have sex with men, lesbian women, and transgender and non-binary individuals.

The article by Zhang et al. examined the intersectional experiences of Asian American sexual minority students in Midwestern universities in the United States. The study employed a qualitative approach, conducting in-depth interviews with nine participants. Thematic analysis was performed to identify patterns of objectification and the internalization of racism, sexism, and heterosexism. The findings suggest that the systemic neglect of intersectional identities has significant implications for students' sense of belonging and psychosocial wellbeing. Based on these results, the authors emphasized the necessity of implementing inclusive policies and institutional reforms to address structural inequities in higher education.

In the final contribution to this Research Topic, Igonya et al. provided an in-depth analysis of forced serial internal displacement trajectories of sexual and gender minorities and their effects on navigating socialites and livelihoods. Qualitative data recalled from 2010 to 2023 were analyzed. Results showed that people belonging to minority groups (e.g., sexuality or gender minority groups) face stigmatization and discrimination at all levels of the socio-ecological model and that, in most cases, grievances and activities against LGBTQ+ people have multiplied, instigating serial forced migration.

In this Research Topic, we have explored several longstanding beliefs and assumptions regarding the effects and consequences of discrimination based on gender or sexuality on wellbeing. The articles have used a wide range of methodological approaches and theoretical frameworks, while focusing on the important effects of the interaction of different types of discrimination on the wellbeing of individuals and society. Most of the articles highlighted how these experiences can have adverse consequences, so it is necessary to delve into the study of them from a holistic perspective of health as a state of wellbeing.

